# A structured approach to design-for-frequency problems using the Cayley-Hamilton theorem

**DOI:** 10.1186/2193-1801-3-272

**Published:** 2014-05-31

**Authors:** Patrick Dumond, Natalie Baddour

**Affiliations:** Department of Mechanical Engineering, University of Ottawa, 161 Louis Pasteur, CBY A205, K1N 6N5 Ottawa, Canada

**Keywords:** Inverse eigenvalue problems, Calyey-Hamilton theorem, Generalized eigenvalue problem, Design-for-frequency, Vibrations

## Abstract

An inverse eigenvalue problem approach to system design is considered. The Cayley-Hamilton theorem is developed for the general case involving the generalized eigenvalue vibration problem. Since many solutions exist for a desired frequency spectrum, a discussion of the required design information and suggestions for including structural constraints are given. An algorithm for solving the inverse eigenvalue design problem using the generalized Cayley-Hamilton theorem is proposed. A method for solving partially described systems is also specified. The Cayley-Hamilton theorem algorithm is shown to be a good design tool for solving inverse eigenvalue problems of mechanical and structural systems.

## 1. Introduction

In mechanical and structural system design, engineers are often faced with the task of designing systems which either have natural frequencies which must fall outside a specific range or operate at exactly certain frequencies. These design problems can be considered as eigenvalue problems, since the eigenvalues are used to determine the natural frequencies (frequency spectrum) of the system. Generally, the problem begins by defining the system’s physical parameters and then calculating the natural frequencies using eigenvalue theory. If specific natural frequencies are sought, empirical or iterative methods are used to modify the system’s physical parameters until the desired eigenvalues are obtained. This approach is both time consuming and indirect. A better approach would be to design the system directly from the natural frequencies.

From a mathematical point of view, this problem is ill-posed. This is because a single set of natural frequencies can be produced by multiple systems and thus multiple solutions are possible. One area that seeks to solve these difficulties and which potentially holds great promise for addressing the problem of design for frequency spectrum is that of inverse eigenvalue problems. Although not currently used for such purpose, the theory could potentially be applied to such design problems. A rather broad field covering many subjects, such as control systems, structural analysis, particle physics and vibrations, inverse eigenvalue problems have an interesting and large field of application. While continuous inverse theories have been studied, such as the classical Sturm-Liouville problem (Chadan 1997; Gel’fand and Levitan 1951; Gantmakher and Kreĭn 2002), a more interesting approach for the purpose of design is to use discrete theory. For the application of inverse eigenvalue theory to the field of vibrations, this would involve the use of discrete matrix representations of real systems. This approach presents greater value since many numerical and analytical tools already exist for the solution of discrete problems and many engineering systems are often modelled as discrete systems.

Much focus has been applied to the study of discrete inverse eigenvalue problems. This has been made clear by a thorough review of the topic by Chu and Golub (Chu 1998; Chu and Golub 2005). Gladwell takes a more direct route in which he considers specific inverse problems and matrix structures related to mechanical vibrations (Gladwell 2004). Particularly, it appears that most of the literature focuses on system identification. One of the most common techniques in inverse eigenvalue problems is to use/measure the system’s spectrum and then constrain the system in some fashion in order to obtain a second spectrum (Hochstadt 1967; Hald 1976; Boley and Golub 1978; de Boor and Saff 1986; Gladwell 1984). This clearly indicates that a system usually exists and that it can be tested to obtain data required for the inverse problem of mathematically reconstructing the system. Although interesting, this approach cannot be used for novel engineering design to construct a system having a specific spectrum without another system on which to base the design.

In most cases, the solution to the inverse problem begins by placing the given desired eigenvalues along the main diagonal entries of a diagonal matrix *Λ*. Additionally, any arbitrary invertible matrix *P* can be used to obtain another solution (matrix) with the same spectrum, namely *PΛP*^− 1^. Since *PΛP*^− 1^ is the trivial solution, pre-conditioning of the *P* matrix is required so that any given structural requirements of the system can be satisfied. Various methods can be used to impose such structure. According to Chu, these methods can be distinguished by the types of procedures used in imposing structure to the matrix (Chu 1998). Structuring matrices by prescribing specific entries has been studied in (Chu 1992; Friedland et al. 1987). Modifying the matrix through the addition of another matrix has also been considered in (Morel 1976; Bohte 1968; Pereyra et al. 1983). Dias de Silva, de Oliveira, and others have studied how multiplying the discrete system by another matrix can affect its structure (Downing and Householder 1956; Dias da Silva 1986; de Oliveira 1972). The use of the well-developed matrix theory for certain structured matrices, such as Jacobi or band matrices, as applied to inverse problems has also been investigated (de Boor and Golub 1978; Erra and Philippe 1997; Biegler-König 1981b; Boley and Golub 1987). Finally, applying least squares methods has shown to be an effective method for finding an approximate solution to inverse eigenvalue problems (Chu and Watterson 1993; Chen and Chu 1996).

In most cases, the research on inverse eigenvalue problems has focused on the existence, uniqueness and computability of a solution. Other studies are typically variations on those described above, including partially described problems where not all spectral information is known. These types of problems have been considered in (Gladwell and Willms 1989; Ram and Elhay 1996), and are of interest for problems requiring only certain frequencies to be specifically determined.

Once an inverse eigenvalue problem has been set up and the type of solution has been chosen, various algorithms can be used to numerically solve the problem. These include orthogonal polynomial methods, the block Lanczos algorithm, the Newton method and the divide and conquer method, as well as several others (de Boor and Golub 1978; Golub and Underwood 1977; Biegler-König 1981a; Gragg and Harrod 1984; Gladwell 1991; Chu 2000). A great deal of research has advanced the field of optimization and has improved our ability to find feasible solutions to various problems. A comprehensive work on the subject is the Encyclopedia of Optimization (Floudas and Pardalos 2009). Although broad in scope, very little has been done to apply inverse eigenvalue theory to actual engineering design problems. In the case of mechanical or structural design, the potential advantage of using such theory when designing a frequency spectrum into a system appears to be immense.

Interestingly, Dias de Silva and de Oliveira have shown that an *n* × *n* matrix always exists when a minimum of *n* − 1 prescribed matrix entries and a prescribed characteristic polynomial are given as design information (de Oliveira 1973; Dias da Silva 1974). Dias de Silva and de Oliveira’s results guarantee existence but not uniqueness of the matrix.

One of the main shortcomings of current inverse eigenvalue theory is the lack of a solution for general matrices having predefined forms but which do not fit within current known solutions. In this paper, a novel approach is considered using the Cayley-Hamilton theorem. The Cayley-Hamilton theorem relates a square matrix over a commutative ring to its characteristic polynomial (Atiyah and Macdonald 1969; Artin 2011). To the authors’ knowledge, the Cayley-Hamilton theorem has not been used as a design tool for inverse eigenvalue problems.

## 2. Problem definition

In this paper, we consider the following problems:

PROBLEM A: *Given a specified frequency spectrum or equivalently a set of eigenvalues, λ*_1_, …, *λ*_*n*_*, construct an nth-order system, described by an n* × *n matrix A, which has λ*_1_, …, *λ*_*n*_*as its eigenvalues.*

Although this problem has been considered for specific forms of matrices (i.e. Jacobi, band or other matrix forms as described above), a general solution approach does not currently exist. Problem A leads into the vibration problem of interest which is also presented here:

PROBLEM B: *Given a specified frequency spectrum or equivalently a set of eigenvalues, λ*_1_, …, *λ*_*n*_*, construct a system with n-degrees of freedom, described by two n* × *n matrices (the mass matrix M and the stiffness matrix K), which has λ*_1_, …, *λ*_*n*_*as its generalized eigenvalues:* det (*K* − *λM*) = 0 *for the given λ*_1_, …, *λ*_*n*_*.*

For an engineer, Problem B relates directly to the design problem stated earlier, where a conservative vibrating system having specific natural frequencies is sought. Finally, a partially described system is considered:

PROBLEM C: *Given a certain number of specified natural frequencies or equivalently eigenvalues, as well as a number of matrix entries, where together there is no less than n pieces of given information, construct an nth-order system, described by an n* × *n matrix A, which has λ*_1_, …, *λ*_*n*_*as its eigenvalues.*

Problem C can be extended to a two matrix problem in the same manner as described for problem B. However, this has not been specifically considered herein.

## 3. Cayley-Hamilton theorem

### 3.1. Basic theory

In order to solve Problem A, a discussion of the Cayley-Hamilton theorem is required. The Cayley-Hamilton theorem states that if *p*(*λ*) is the characteristic polynomial of a square matrix *A*, obtained from *p*(*λ*) = det (*λI* − *A*), then substituting *A* for *λ* in the polynomial gives the zero matrix. Thus, by applying the theorem, matrix *A* satisfies its own characteristic polynomial, *p*(*A*) = 0 (Knapp 2006).

The Cayley-Hamilton theorem can be useful in inverse eigenvalue problems beyond the typical statement that a square matrix satisfies its own characteristic equation. Once the characteristic polynomial of a system is found from desired spectral data, the Cayley-Hamilton theorem can be used to find an unknown matrix *A*, which represents the system. A set of unknown entries of the matrix *A* can be solved from the set of equations that arise from the Cayley-Hamilton theorem. For example, suppose that a 2 × 2 matrix *A* has the form1

In order to solve the inverse eigenvalue problem we must populate the entries of matrix *A* by using the Cayley-Hamilton theorem and the set of desired eigenvalues. In order to visualize the process, suppose that the desired eigenvalues are -2 and -3. The characteristic polynomial is then constructed as2

Using the Cayley-Hamilton theorem, *λ* is replaced by *A* of equation (1) in equation (2), such that3

where *I* is the identity matrix. Expanding equation (3) gives us four equations with four unknowns:4

However, equation independence is unclear. A discussion is found in Section 4.1. Solving equation (4) leads to two dependent solutions:5

At this point, any values can be assigned to *a*_21_ and *a*_22_ and the matrix *A* will have the desired eigenvalues given in equation (2). Consequently, Problem A has been solved, although it is clear that many solutions exist. This solution is particularly useful in solving inverse eigenvalue problems as it gives a range of *A* matrix values for which a system produces the same eigenvalues. The limiting factors are then based on the physical limits and fixed parameters of the system.

### 3.2. Generalized Cayley-Hamilton theorem for mass and stiffness matrices

Problem B is related to Problem A, but takes on a more general form which is conducive to real physical systems. When considering a conservative vibrating system, the factors that control its frequency spectrum are the system’s mass and stiffness. Typically, continuous systems are discretized in order to simplify the analysis. By doing so, the system is described using mass (*M*) and stiffness (*K*) matrices. The forward generalized eigenvalue problem involves solving the equation det (*K* − *λM*) = 0 for the system’s generalized eigenvalues, *λ*. Although the characteristic polynomial is similar to the single matrix case, it now involves two matrices rather than one. Therefore, the lesser-known generalized Cayley-Hamilton theorem must be used (Chang and Chen 1992).

The generalized Cayley-Hamilton theorem is modified to include a second square matrix *B*. The characteristic polynomial takes the form *p*(*λ*) = det (*A* − *λB*). By substituting *A* and *B* for *λ* into the characteristic polynomial a similar relationship is satisfied,6

where *c*_*n*_ is the coefficient of *λ*^*n*^ in *p*(*λ*). Equation (6) is valid as long as *B* is non-singular.

If matrices *A* and *B* commute (i.e. *AB* = *BA*), then the generalized Cayley-Hamilton theorem can be written as7

where no other restrictions are placed on matrix *B*.

For the generalized eigenvalue problem of a vibrating system, the characteristic equation of the system is obtained by calculating the determinant of (*K* − *λM*). Thus, the Cayley-Hamilton theorem for a conservative vibrating system is obtained by replacing the matrix *B* with the mass matrix *M* and the *A* matrix by the stiffness matrix *K* in equations (6) and (7). Solving these equations leads to the solution of Problem B.

### 3.3. Numerical example

Once again, a numerical example is used to demonstrate concepts. Using the same eigenvalues as for the previous example gives the characteristic polynomial of equation (2). This time, the modified generalized Cayley-Hamilton theorem is used to form an equation for unknown matrices *K* and *M* such that8

Assuming the *K* and *M* matrices have a similar form to that of *A* in equation (1), then equation (8) can be expanded to give four equations with eight unknowns. Solving these equations produces several results, one of which can be written as9

Once again it becomes clear that only 2 solutions are dependent, and selecting any values for *k*_12_, *m*_12_, *k*_21_, *m*_21_, *k*_22_, *m*_22_, will result in *K* and *M* matrices that produce a solution with the desired eigenvalues.

Increasing the size of the square matrices increases the number of independent variables quicker than it does the dependent variables. In other words, for an *n*-th order system with *n* specified eigenvalues (natural frequencies), 2*n*^2^ unknown variables are required to find *K* and *M*, but as will be shown later in this paper, the generalized Cayley-Hamilton theorem only produces *n* independent equations.

### 3.4. Spring-mass system example

**Figure 1:**
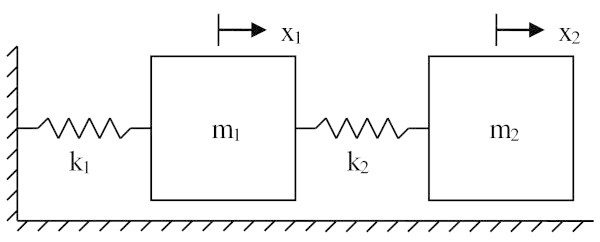
**2DOF spring-mass system.**

Using a typical 2 degree-of-freedom (2DOF) spring-mass system, as seen in Figure 1, many properties of the method can be explored. Although simple, this problem allows us to see the basics of how the method works. A more complicated problem is included as an appendix for further reading.

In order to use the Cayley-Hamilton inverse method, the structure of the system must first be explored in the forward sense. In essence we must pre-condition the mass and stiffness matrices to account for the system’s physical constraints. The equation of motion of the system can be written as10

where  is the displacement vector and *M* and *K* are the mass and stiffness matrices respectively. The goal of the approach is: given the system eigenvalues, *λ*_1_ and *λ*_2_, determine the *M* and *K* matrices that give rise to these eigenvalues. Determining the matrices *M* and *K* in accordance with relevant system constraints would be the engineering design problem.

Once the structure of the system has been set, the inverse solution can begin. First, from the set of desired eigenvalues, *λ*_1_ and *λ*_2_, a characteristic polynomial is formed such that11

Using the Cayley-Hamilton theorem with *M*, *K* as given in equation (10), along with the desired characteristic equation (11), a set of four equation and four unknowns is found:12

A solution is obtained as13

In this case, choosing any value for two of the four variables will lead to a solution which satisfies the characteristic polynomial and thus has the given natural frequencies/eigenvalues. This solution is the same as that presented by Gladwell in (Gladwell 2004), although the method of obtaining this solution is different.

## 4. Analysis

### 4.1. Information produced by the Cayley-Hamilton theorem

We show here that given *n* distinct eigenvalues for an *n*-th order system, the Cayley-Hamilton theorem can produce at most *n* independent equations, even though *n*^2^ equations are produced. Let *p*(*t*) = *c*_*n*_*t*^*n*^ + *c*_*n*−1_*t*^*n*−1^ + …*c*_1_*t* + *c*_0_ be the characteristic polynomial of an *n* × *n* matrix *A*. The Cayley-Hamilton theorem states that *p*(*A*) = *c*_*n*_*A*^*n*^ + *c*_*n*−1_*A*^*n*−1^ + …*c*_1_*A* + *c*_0_ is the zero matrix. Suppose that the matrix *A* is diagonal and let the diagonal entries be *λ*_1_, *λ*_2_, …, *λ*_*n*_. The characteristic polynomial is14

In this case, *p*(*A*) is also a diagonal matrix with exactly *n* equations. In fact, the i-th diagonal entry is *p*(*λ*_*i*_).

Now consider the case that *A* is not diagonal. Given *n* distinct eigenvalues for *A*, then *A* is diagonalizable, so that *A*’ = *P*^−1^*AP* is diagonal for some invertible matrix *P*. The characteristic polynomial of *A*’ is the same as the characteristic polynomial *p*(*t*) of *A*. In fact, the diagonal matrix *A*’ is the trivial solution to our design problem and the ‘structure’ of the design problem is housed in the matrix *P.* It is known that (Artin 2011)15

Equation (15) states that *p*(*A*) can be obtained by combining the equations contained in *p*(*A*’). At the same time, since *A*’ is itself diagonal, then matrix *p*(*A*’) contains exactly *n* equations. Thus, this states that *p*(*A*) is a combination of exactly *n* independent equations and we can expect that although *p*(*A*) has *n*^2^ equations, only *n* of them are independent.

It is also important to point out that the *n* independent equations obtained from the Cayley-Hamilton theorem are each *n*-th order polynomials. For a polynomial system with *n* unknowns and also *n* equations, then Bézout’s theorem states that our problem has *n*^*n*^ complex solutions (Coolidge 1959).

### 4.2. Required design information

It is clear that the spectral information (eigenvalues) alone is not enough information to complete a design. The method presented, along with all other methods, is limited by the fact that an *n*-th order system can produce at most *n* independent equations, even though *n*^2^ equations are obtained via applying the generalized Cayley-Hamilton theorem, as discussed above. If the matrices are completely unknown then there may be as many as 2*n*^2^ unknown entries in the mass and stiffness matrices. The Cayley-Hamilton method can produce at most *n* independent variables and the remaining equations must be specified in other ways. For creating physically realistic systems, this generally entails pre-conditioning or constraining the structure of the matrices.

For solving discrete conservative vibration problems, Equation (6) can be used as long as the *M* matrix is non-singular and equation (7) can be used if *M* and *K* commute. It becomes very clear by considering equation (9) that more information is required in order to build a suitable vibrating system based on mass and stiffness. From an engineer’s point of view, any system which can produce similar eigenvalues has the potential of being suitable, as long as it fits within the physical criteria set at the outset of the project. It is obvious that equation (9) can produce an infinite number of possible solutions. Equation (9) represents only one of several solutions to the equation presented in (8). As the system’s order increases, so does the number of potential solutions (Bézout’s theorem). Thus, engineers have many solutions at their disposal for creating suitable and optimized designs.

It is then evident why many engineers develop and adopt their own unique methods for creating and optimizing designs. These represent but one of many possible solutions. Evidence tends to contradict arguments that there is only one approach to design or only one solution to the problem.

The inverse problem is then not limited by the eigenvalues, and in fact the eigenvalues alone do not contain enough information from which to build a physical system. Therefore, other information is required in order to complete the design.

### 4.3. Structural constraints

One of the easiest ways to limit the number of matrix entries and include structural constraints is by including zero entries or by incorporating symmetry into the matrices. These constraints are interesting because they follow directly from real systems. Taking as an example the 2DOF spring-mass system developed in Section 3.4, it can be seen that only diagonal entries are present in the mass matrix and the stiffness matrix is made symmetric by forcing off-diagonal terms to be the same. Physically, the structure of these matrices reflects the fact that the two degrees of freedom of the system are coupled via a stiffness element coupling. It can be seen in Figure 1 that the masses are connected via springs (stiffness elements) only. Furthermore, the symmetry of the stiffness matrix is a consequence of Newton’s third law. The system is further constrained by the fact that the off-diagonal terms in the stiffness matrix are not independent, but are related to the diagonal terms, once again reducing the number of unknowns. In this case, incorporating physical constraints into the mathematical structure of the problem has reduced the number of unknowns from 2*n*^2^ to just *n*^2^.

In this case, the engineer still has the freedom to choose from several systems which would satisfy the requirements. Further constraints may come in the form of available components such as stiffeners which must fit within specified physical dimensions or the financial budget.

From this stems the importance of the forward problem. Continuous systems are typically discretized using various methods which produce a model of the system in matrix form. The form of the matrix is heavily dependent on the choice of discretization method. The inverse problem is consequently affected since it seeks to create a matrix that matches the form as stipulated at the outset. Although the Cayley-Hamilton theorem does not discriminate in its ability to solve these various matrix forms, it is possible that certain discretization methods lead to simpler forms or matrices containing fewer variables. In this sense, the design may be easier to fully define.

### 4.4 Partially described systems

Another aspect that affects the amount of design information required is the information available for the design. Thus far, the entire spectral set has been used as design information, as well as specification of matrix entries when necessary. However, as stated in (Chu 1998), often only portions of the entire spectrum are available. This is termed a partially-described inverse eigenvalue problem. This is true whether it is the information stipulated via the design requirements or whether it is the experimental data available for system identification. Regardless of the information available, the Cayley-Hamilton theorem can be used to produce *n* pieces of information for an *n*-th order system stemming from *n* degrees of freedom. If certain eigenvalues are missing, the Cayley-Hamilton theorem can still be used.

Typically, in order to completely solve an *n*-th order inverse problem using the Cayley-Hamilton theorem, only *n* unknown values should be present in the problem, regardless of their appearance along the solution path. Problem C makes full use of this detail during its solution by pre-conditioning the matrix being solved to account for this. For a 3DOF system then, only three unknowns should be present. Consider for example the following system which has three known eigenvalues *λ*_1_ = 0.47, *λ*_2_ = 4.66, *λ*_3_ = 10.87 and three unknown entries in the matrix16

Using the Cayley-Hamilton theorem as described in Section 3, the matrix *A* can be determined such that *a*_1_ = 9.43, *a*_2_ = 1.23, *a*_3_ = 2.06 which gives a matrix solution for the system of17

As in Problem C, it is often the case that only partial spectral information is available. Therefore, the Cayley-Hamilton theorem can be used if the amount of missing spectral information is replaced by the same amount of matrix entry information. So, if only *λ*_3_ = 10.87 is known then two of the three *a* matrix entries must be known. If *a*_2_ = 1.23 and *a*_3_ = 2.06 are known in the example above, then solving the Cayley-Hamilton equations gives *a*_1_ = 9.43, *λ*_1_ = 0.47, *λ*_2_ = 4.66. Consequently, solving Problem C is not much different from solving Problem A.

The same is true if the system is made up of a mass matrix *M* and a stiffness matrix *K*, except in this case, the generalized Cayley-Hamilton theorem must be used.

The question then becomes what is the better strategy for design? Is it better to specify the entire spectrum even though only a select few eigenvalues are critical? In this case, the analysis would lead to a full solution of the matrix if any real solutions are possible based on the prescribed matrix form. Or is it better to only specify the critical eigenvalues and solve for the remainder by specifying more information in the matrix? In this case, an alternate method for determining this extra matrix information would be required. From experience, it would appear that the latter method is generally easier given the need to produce a real system, especially since not all spectra produce real systems. The main difficulty appears to be in setting up the matrix form. This is achieved by looking at the forward solution method. In most cases, the forward solution will utilize some form of discretization, be it finite elements, global elements, finite difference or other methods. The method chosen has a large impact on the structure of the matrix, making the solution easier or more difficult depending on the situation. Also, in discretizing, the method chosen to relate material parameters has a large effect on the number of independent variables. Therefore it is extremely important to ensure that simplification methods are properly considered.

## 5. Discussion

### 5.1 Implementation

The implementation of the Cayley-Hamilton theorem is particularly suited to symbolic computer algebra systems such as Maple, Mathematica or Mathcad since the problem can be efficiently set up within the software. The characteristic polynomial can be directly created from the eigenvalues, and subsequently the Cayley-Hamilton theorem equation can be constructed in order to produce the set of governing equations, finally the equations can be solved (in some cases analytically) in order to populate the given matrices. If the system is 5-th order or higher, it may be easier to use a minimization procedure such as the DirectSearch package available in Maple to simplify computation.

On the other hand, if a solution to the set of governing equations is all that is sought; numerical solvers such as MATLAB are particularly well-suited for obtaining numerical solutions.

### 5.2. Non-physical solutions

Like most design tools, the Cayley-Hamilton theorem is not without drawbacks. Certain aspects of the theorem must be diligently considered by the engineer in order to ensure proper solution compliance.

It is of utmost importance that the system matrices be constructed from physical knowledge. Although a solution is possible with the Cayley-Hamilton theorem, it may not always produce real entries in the matrices. Since the goal is to reproduce a real system, complex matrix entries do not satisfy the design goal.

It is well known, that the roots of the polynomials are sensitive to perturbations of the coefficients (Chu 1998), therefore polynomials constructed this way are usually easily subject to errors. This is especially true for system identification, where experimental data is quite often inexact. However, since frequencies specified in design (that is, the desired frequencies of vibration) are generally obtained from extensive experimentation or through other means all together, the effect of perturbations is significantly lessened.

### 5.3 Algorithm

In order to use the Cayley-Hamilton theorem as a tool for the design of an *n* dimensional vibrating system based on knowledge of desired natural frequencies and/or physical parameters, 2*n*^2^ − *n* additional pieces of design information are required in addition to the *n* desired natural frequencies. An algorithm for solving Problem B is as follows:

ALGORITHM. *Given the n desired eigenvalues λ*_1_, …, *λ*_*n*_*of an n-th order vibrating system*

1. *Generate the characteristic polynomial:*
2. *Generate the M and K matrices from physical parameters and by using the* 2*n*^2^ − *n pieces of design information, applying symmetry and any other techniques based on the discretized forward problem and leaving an n number of unknowns;*
3. *Generate the Cayley-Hamilton theorem equation:*
  *or for commuting M and K matrices:*
4. *Extract n*^*2*^*equations from the Cayley-Hamilton equation in 3;*
5. *Select n non-zero independent equations (the Cayley-Hamilton matrix diagonal entries work well);*
6. *Compute the n unknowns by solving the n independent equations;*
7. *Insert the n computed values into their appropriate places in the M and K matrices;*
8. *Verify that a valid solution is obtained by calculating* det (*λM* + *K*) = 0 *and ensuring that the initially given eigenvalues are obtained.*

*The output consists of n matrix entries of the M and K matrices.*

A similar algorithm can be applied to Problems A and C. In the case of Problem C, *n* − *m* eigenvalues are given, as well as *m* matrix entries, where *m* is the number of unknown or unspecified eigenvalues. The output consists of *m* eigenvalues and the remainder of the unknown matrix entries.

## 6. Conclusion

In this paper, we considered a tool that can be used for discrete design-for-frequency engineering problems. An engineer would generally prefer a direct approach to design-for-frequency when designing a mechanical or structural system, rather than a heuristic trial-by-error approach. In this paper, we showed that the Cayley-Hamilton theorem can be a good design tool for achieving this. Unlike other methods, this approach is not limited in application to any specific type of matrix structure. Although many mathematical solutions usually exist, only a finite number of solutions are actually physically valid. This does not, however, depend on the eigenvalues, but rather on the dimensions, the physical requirements and the structure of the system, which dictate many of its parameters. The Cayley-Hamilton theorem allows the easy inclusion of these extra parameters in order to get a physically realistic design without iteration.

Regardless of the specified design information for an *n*-th order system, *n*^2^ − *n* (or 2*n*^2^ − *n* for the general case) additional pieces of information are required as input in addition to the *n* desired eigenvalues, in order to completely solve the inverse eigenvalue problem and hence design the system. This follows since the Cayley-Hamilton theorem can supply at most *n* pieces of information to the design, as shown in this paper. The source of the information, whether eigenvalues or matrix entries, is of little significance. Hence, establishing the forward equations of motion in order to pre-condition the discrete matrix system structure based on the physical system of interest is emphasized.

## 7. Appendix - example of using the Cayley Hamilton inverse method for brace design

### 7.1 Problem statement

Given a desired fundamental frequency, construct a brace-plate system as described by a mass matrix *M* and a stiffness matrix *K*. All dimensional (geometric) properties of the brace-plate system are assumed to be specified and fixed except for the thickness of the brace *h*_*c*_, the design variable for which we must solve.

### 7.2 Forward model

**Figure 2:**
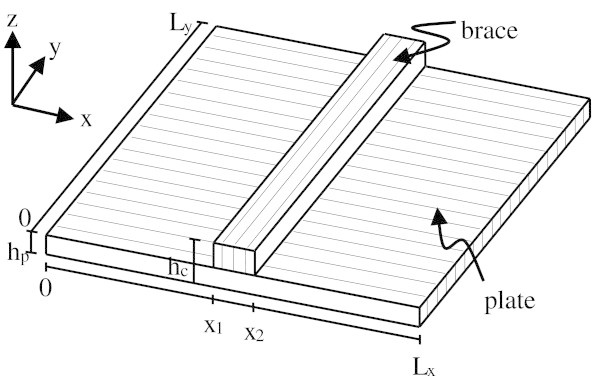
**Orthotropic plate reinforced with a rectangular brace.**

The model is based on an orthotropic plate structurally reinforced by a brace in the weaker plate direction. The model is shown in Figure 2.

The forward model is discretized using the assumed shape method. The assumed shape method is an energy method which uses global plate elements within the kinetic and strain energy plate equations in order to determine the system’s equations of motion, from which the mass and stiffness matrices are extracted (Meirovitch 1996). For the details of the development of the large mass and stiffness matrices, the reader is referred to (Dumond and Baddour 2013). The system is assumed simply supported, conservative and the material properties are assumed orthotropic. The forward model is created assuming the mechanical properties are all related to Young’s Moduli in the *y*-direction.

### 7.3 Inverse model

The goal is to reconstruct the brace-plate system from a desired fundamental frequency. The generalized Cayley-Hamilton theorem inverse eigenvalue method is used as explained in Section 3.2.

**Figure 3:**
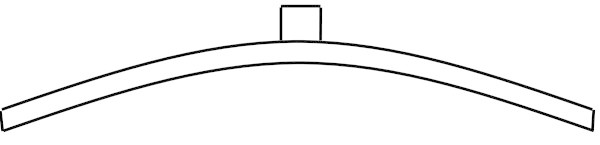
**Cross section of the brace-plate system’s fundamental modeshape.**

A cross section of the fundamental modeshape is shown in Figure 3. It is clear that the brace affects the maximum amplitude of this modeshape, thus also affecting the associated frequency. In order to adjust the fundamental frequency of the brace-plate system to a desired value, it is necessary to adjust the thickness of the brace (Dumond and Baddour 2012).

### 7.4 Modeling considerations

Since the mechanical properties vary based on *E*_*y*_, and that the brace thickness controls the brace-plate system’s fundamental frequency, the forward model is created using the assumed shape method while leaving these two parameters as variables. Thus, the mass matrix *M* is a function of *h*_*c*_, the height of the brace-plate system at the (assumed fixed) location of the brace, and the stiffness matrix *K* is a function of *h*_*c*_ and also *E*_*y*_. Here, we use 2 × 2 trial functions in the assumed shape method. Hence, 4^th^ order square matrices are created. The trial functions used are those of the simply supported rectangular plate such that18

where *m* are the modal numbers, *q* the time function and *w* is the displacement variable normal to the plate. The displacement variable *w* is then used directly in creating the kinetic and strain energy equations of the simply supported rectangular plate. These equations are broken into three sections as shown in Figure 2 in order to take into account the brace. This procedure is well described in (Dumond and Baddour 2013). It is assumed that the *Ey* is known and used as input information into the stiffness matrix. This leaves *h*_*c*_ as the only unknown parameter, appearing in both the mass and stiffness matrices.

In order to solve these matrices from the desired fundamental frequency, we must first create the characteristic polynomial using the desired frequency,19

where *a* is the desired frequency and *b*_*1*_*-b*_*3*_ are unknown values which need to be found. Since we have assumed 2 × 2 trial functions so that the mass and stiffness matrices are both 4 × 4, the characteristic polynomial must be fourth order, as shown in equation (19). Subsequently, *p(λ)* is expanded so that the polynomials coefficients can be found. Once the polynomial is created, the Cayley-Hamilton equation can be written by substituting *(M*^*-1*^ *K)* for *λ* into equation (19):20

where *c*_*n*_ are the coefficients of *λ* in *p(λ)* determined via equation (19). Equation (20) produces sixteen equations, of which only four are independent. Solving the equations on the main diagonal for the four unknowns (*h*_*b*_, *b*_*1*_, *b*_*2*_, *b*_*3*_) produces 4^4^ = 256 possible solutions, according to Bézout’s theorem. From the set of all possible solutions, complex solutions can be immediately eliminated as not being physically meaningful. Clearly, further constraints must be added to the solution in order to get a solution which fits within the desired physical limits. These physical limits are based on the maximum and minimum brace dimensions which are required to compensate for the range of plate stiffnesses used during the analysis, as well as the range of natural frequencies which can be obtained using these system dimensions. Thus, the following constraints are implemented into the solution:21

Solving the four equations obtained from equation (20) within the constraints provided by (21) yields a physically realistic solution which satisfies the desired fundamental frequency, as well as the system’s parameters.

### 7.5 Results

The material properties used during the analysis are given in Table 1.

**Table 1:** **Material properties**

Material properties	Values
Density – *μ* (kg/m^3^)	403.2
Young’s modulus – *E*_*y*_ (MPa)	850
Young’s modulus – *E*_*x*_ (MPa)	*E*_*y*_ */*0.078
Shear modulus – *G*_*xy*_ (MPa)	*E*_*x*_ *×* 0.064
Poisson’s ratio – *ν*_*xy*_	0.372
Poisson’s ratio – *ν*_*yx*_	*ν*_*xy*_ × *E*_*y*_/*E*_*x*_

The dimensions used for the model throughout the analysis of the brace-plate system are shown in Table 2.

**Table 2:** **Dimensions of brace-plate model**

Dimensions	Values
Length – *L*_*x*_ (m)	0.24
Length – *L*_*y*_ (m)	0.18
Length – *L*_*b*_ (m)	0.012
Reference – *x*_*1*_ (m)	*L*_*x*_/2 – *L*_*b*_/2
Reference – *x*_*2*_ (m)	*x*_*1*_ + *L*_*b*_
Thickness – *h*_*p*_ (m)	0.003
Thickness – *h*_*b*_ (m)	0.012
Thickness – *h*_*c*_ (m)	*h*_*p*_ + *h*_*b*_

These dimensions refer to those shown in Figure 2, where ‘*p*’ refers to the plate’s dimensions, ‘*b*’ refers to the brace’s dimensions and ‘*c*’ refers to the dimensions of the combined system.

As a basis for comparison, a plate having a Youngs’ moduli of *E*_*y*_ = 850 MPa to which a brace is attached with a combined brace-plate thickness of *h*_*c*_ = 0.015 m is found to have a fundamental natural frequency of 687 Hz, calculated using the forward model. The analysis is then performed using the inverse method described in the previous section. As *E*_*y*_ of the plate is varied, the thickness of the brace-plate section is calculated such that the fundamental frequency of the brace-plate system is kept consistent at 687 Hz. The results of the computations can be found in Table 3.

**Table 3:** **Results of the inverse model analysis**

Young’s modulus ***E***_***y***_(MPa)	Brace thickness ***h***_***c***_(m)	Fundamental frequency ***a*** (Hz)
750	0.01576	687
800	0.01536	687
813	0.01527	687
850	0.01500	687
900	0.01466	687
950	0.01435	687

Clearly, adjusting the thickness of the brace also has an effect on the other natural frequencies. These can be seen in Table 4.

**Table 4:** **Calculated frequencies of the inverse model analysis**

Young’s modulus ***E***_***y***_(MPa)	Brace thickness ***h***_***c***_(m)	***b***_***1***_(Hz)	***b***_***2***_(Hz)	***b***_***3***_(Hz)
750	0.01576	774	1360	2653
800	0.01536	782	1363	2650
813	0.01527	784	1364	2650
850	0.01500	790	1366	2648
900	0.01466	798	1370	2645
950	0.01435	806	1374	2642

Interestingly, the constraints indicated in equation (21), although physically strict, allow for more than one solution in certain cases. An example is shown in Table 5.

**Table 5:** **Alternate brace thickness solution satisfying the physical constraints**

Young’s modulus ***E***_***y***_(MPa)	Brace thickness ***h***_***c***_(m)	***a*** (Hz)	***b***_***1***_(Hz)	***b***_***2***_(Hz)	***b***_***3***_(Hz)
750	0.01359	687	570	1149	2185

### 7.6 Discussion

From these results, it is evident that designing a brace-plate system starting with a desired fundamental frequency, and using the proposed Cayley-Hamilton method, is possible. Table 3 clearly shows that by adjusting the thickness of the brace by small increments (10^-5^ m, machine limit), it is possible to compensate for the variation in the cross-fibre stiffness (*E*_*y*_) of the plate so that the fundamental frequency of the combined system is equal to that of the benchmark value of 687 Hz. The results obtained using the Cayley-Hamilton theorem algorithm match those values obtained using the forward model exactly. However since no account has been taken of the other frequencies during the analysis, Table 4 shows that frequencies *b*_*1*_ to *b*_*3*_ vary considerably from those values obtained for *E*_*y*_ = 850 MPa. Therefore, it is important to ensure that there is a good understanding of what your model can control. Moreover, it is interesting to note that within the strict physical constraints of (21), there is more than one brace-plate system (solution) that satisfies the Cayley-Hamilton theorem of equation (20). From Table 5 it can be seen that an alternate solution to the system exists, different from the one presented in Table 3, for a plate having a *E*_*y*_ of 750 MPa. In this case, by reducing the thickness of the brace, it is still possible to achieve a system having the desired frequency of 687 Hz. However, the desired frequency is no longer the fundamental frequency but rather becomes the second frequency and the fundamental has been replaced with a fundamental frequency of 570 Hz. It is important to keep this phenomenon in mind while designing a system. This is especially true if the order in the spectrum of a certain frequency associated with a certain modeshape is absolutely critical.
